# Efficient high repetition rate electro-optic Q-switched laser with an optically active langasite crystal

**DOI:** 10.1038/srep30517

**Published:** 2016-07-27

**Authors:** Shihui Ma, Haohai Yu, Huaijin Zhang, Xuekun Han, Qingming Lu, Changqin Ma, Robert I. Boughton, Jiyang Wang

**Affiliations:** 1 State Key Laboratory of Crystal Materials and Institute of Crystal Materials, Shandong University, Jinan 250100, China; 2 Chemistry and Chemical Engineering, Shandong University, Jinan 250100, China; 3 Department of Physics and Astronomy, Green State University, Bowling Green, OH 43403, USA

## Abstract

With an optically active langasite (LGS) crystal as the electro-optic Q-switch, we demonstrate an efficient Q-switched laser with a repetition rate of 200 kHz. Based on the theoretical analysis of the interaction between optical activity and electro-optic property, the optical activity of the crystal has no influence on the birefringence during Q-switching if the quarter wave plate used was rotated to align with the polarization direction. With a Nd:LuVO_4_ crystal possessing a large emission cross-section and a short fluorescence lifetime as the gain medium, a stable LGS Q-switched laser was designed with average output power of 4.39 W, corresponding to a slope efficiency of 29.4% and with a minimum pulse width of 5.1 ns. This work represents the highest repetition rate achieved so far in a LGS Q-switched laser and it can provide a practical Q-switched laser with a tunable high repetition rates for many applications, such as materials processing, laser ranging, medicine, military applications, biomacromolecule materials, remote sensing, etc.

High repetition rate pulsed lasers with high peak power have wide applications in materials processing, laser ranging, medicine, military applications, biomacromolecule materials, remote sensing, etc.^1^,^2^,^3^,^4^,^5^. For instance, in materials processing, the processing speed depends upon the repetition rate of the laser source. High repetition rate operation can be achieved by passive Q-switching^6^ or active Q-switching. Compared with passive Q-switching, active Q-switching exhibits a stable pulse energy and low temporal jitter at the repetition rate^7^. Active Q-switching mainly utilizes two common Q-switch modes, the acousto-optic (AO)^8^,^9^ and the electro-optic (EO) Q-switching^10^. The AO Q-switching is limited for applications to many fields with high repetition rates because of its tendency to produce a long pulse^7^. Compared with the AO mode, the EO Q-switching mode has the advantages of faster switching, better hold-off ability^7^, larger pulse energy and a controllable repetition rates^5^,^11^. Electro-optic crystals with a large electro-optic coefficient, broadband transmission spectral range, high optical damage threshold and ease of achieving large size are favorable for the application of electro-optic Q-switching^9^. However, up to now the ideal electro-optic crystals have not been found, although many electro-optic crystals have been developed. The most widely used electro-optic crystals comprise KD_2_PO_4_ (KD^*^P), LiNbO_3_ (LN), β-BaB_2_O_4_ (BBO) and Rb_6_TiOPO_4_ (RTP)^12^ which nevertheless have some intrinsic problems, such the deliquescence of KD^*^P, relatively low optical damage threshold of LN (100 MW/cm^2^)^13^, the low-symmetry structure and natural birefringence of RTP^14^ and the difficulty of achieving large-size crystal growth of BBO^15^. Besides, high repetition rate pulsed output is constrained by the piezoelectric ring effect^16^,^17^, e.g. KD^*^P can only be used at repetition rate under 10 kHz^18^. Based on the above considerations, the present limitations of existing electro-optic crystals have constrained the development and applications of electro-optic Q-switched lasers, especially those with high repetition rates.

Recently, a multi-functional langasite (LGS) crystal has attracted a great deal of research attention for electro-optics applications. It has an electro-optic coefficient of 2.3 × 10^−12^ m/V^19^, a high optical damage threshold which is 950 MW/cm^2 ^19 and a broadband transmission spectrum^20^. However, LGS exhibits optical activity, which means that the light polarization direction is rotated as it propagates through the crystal. It has been determined that the effect of optical activity on the electro-optic process can be eliminated by reversing the effect, which means that light propagating along the optic Z-axis should be reversed to propagate along the Z-axis. Relying on its broadband transmission spectrum and electro-optic properties, a LGS Q-switched laser with the wavelength ranging from 1.06 μm to 2.79 μm was reported^21^,^22^,^23^. A LGS Q-switched laser with 30 kHz^21^ repetition rate was also constructed. Since it has a small piezoelectric coefficient of 6 × 10^−12^ C/N^24^, less than a quarter of that of BBO, motivation is provided to investigate LGS Q-switching in high repetition rate lasers. Based on Q-switching theory, the pulse width and peak power are dependent on the cavity length, which indicates that in LGS Q-switching based on the reversibility of optical activity would generate a larger pulse width, in spite of the design complications encountered with the laser cavity. Although LGS Q-switched lasers with a rotated quarter wave plate or polarizer have been reported, there is no analysis on the mechanism supporting the application of this technique. In this work, we theoretically analyze the coupling between optical activity and the electro-optic properties of LGS during the Q-switching process and find that the only effect of optical activity is a rotation along the propagating direction, which can be eliminated simply by rotating the quarter wave plate. Based on the theoretical analysis, we constructed a LGS Q-switched laser in experiments with a Nd:LuVO_4_ crystal which has a large emission cross-section and a short fluorescence lifetime^25^,^26^. This work results in the operation of a laser with a 200 kHz repetition rate and a 5.1 ns pulse width, which represent the highest repetition rate and narrowest pulse width in Q-switched LGS crystal lasers.

## Results

**Figure 1:**
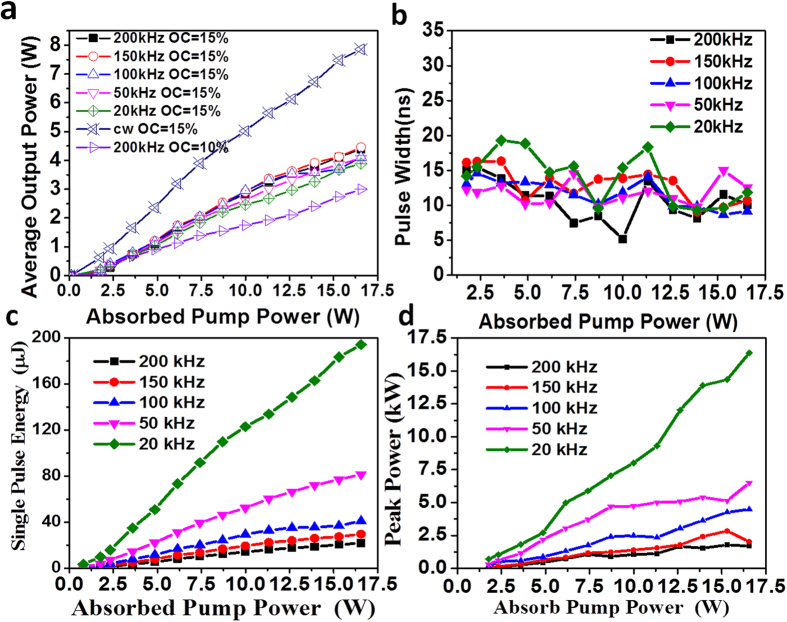
The performances of cw and Q-switched pulsed lasers. (**a**) Output power versus absorbed pump power of cw and Q-switched pulsed lasers. (**b**) Single pulse width vs. absorbed pump power of Q-switched pulsed laser with OC = 15%. (**c**) Single pulse energy vs. absorbed pump power at different repetition rates with OC = 15%. (**d**) Peak power vs. absorbed pump power of Q-switched pulsed laser with OC = 15%.

Using a two-mirror laser cavity, we studied the Q-switched laser performance. Upon removing the polarizer, LGS Q-switch and QWP, a continuous wave (cw) laser was obtained and the results were shown in Fig. 1(a). The optimized cw output power was achieved using an output coupler with a transmittance of 15%. Under an absorbed pump power of 16.56 W, the maximum output power of 7.86 W was achieved with a conversion efficiency of 47.5% and a threshold of 0.12 W. The output power linearly increases with the absorbed pump power when the slope efficiency was 50%. Using a polarizer, the output laser was identified to be π-polarized.

By inserting the polarizer, Q-switch and QWP into the cavity, a Q-switched laser is obtained by applying a driving voltage. A plot of the output power vs. the absorbed pump power with repetition rates ranging from 20 kHz to 200 kHz is presented in Fig. 1(a). From this figure, it is clear that the output power is dependent on the repetition rate and increases both as an increases of the absorbed pump power and repetition rate. With an absorbed pump power of 16.5 W, the maximum output power was measured to be 3.89 W, 4.08 W, 4.11 W, 4.46 W and 4.4 W at a repetition rate of 20 kHz, 50 kHz, 100 kHz, 150 kHz and 200 kHz, respectively. We also find that the output power is comparable at repetition rates of 150 kHz and 200 kHz, which indicates that the loss generated by the LGS switching effect for a repetition rate above 150 kHz saturates the output power. By comparison, an output coupler with a transmittance of 10% was also employed for the 200 kHz repetition rate and results are shown in Fig. 1(a) with a maximum output power of 3 W.

**Figure 2:**
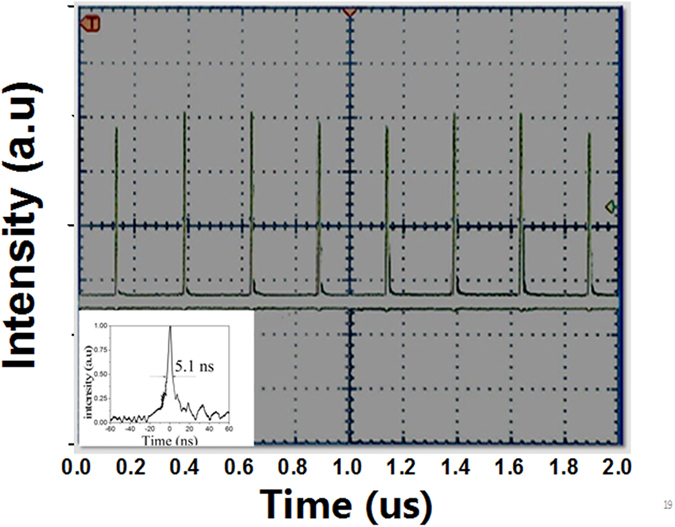
Pulse train with a repetition rate of 200 kHz. Inset: Pulse profile with shortest pulse width of 5.1 ns.

The pulse behavior with a transmittance of 15% was recorded with a digital oscilloscope. The variation of the pulse width with absorbed pump power is shown in Fig. 1(b). From this figure, it can be seen that the pulse width ranges from 19. 3 ns to 5.1 ns with different absorbed pump powers and repetition rates. There is no obvious relationship between the pulse width and the absorbed pump power at the same repetition rate. The 5.1 ns pulse profile achieved with an absorbed pump power of 10 W and repetition rate of 200 kHz is shown in the inset of Fig. 2. The pulse train obtained at a repetition rate of 200 kHz is shown in Fig. 2, which indicates that the pulse strength is stable with a variation of only 6.2% without any piezoelectric ringing effect. These results indicate that the LGS crystal can be used as a high repetition rate Q-switch operating at as high as 200 kHz. Using average output power and repetition rate, the pulse energy was calculated and the plots of pulse energy versus absorbed pump power at various frequency rates are shown in Fig. 1(c). The pulse energy decreases with repetition rate and linearly increases with absorbed pump power. The maximum pulse energy is 194 μJ at a repetition rate of 20 kHz and the maximum pulse energy at the repetition rate of 200 kHz is 22 μJ with an absorbed pump power of 16.5 W. With the pulse energy and pulse width, the peak power can be calculated. The maximum peak power also decreases with repetition rate and linearly increases with absorbed pump power, its plots are shown in Fig. 1(d). The maximum peak power is 16.4 kW and 1.78 kW for a repetition rate of 20 kHz and 200 kHz, respectively.

## Discussions

It should be noted that there was no damage observed in any component of the laser cavity. To sum up the present results, we demonstrated Q-switched performances at different repetition rates as detailed in Table 1. The present results represent the highest repetition rate observed so far in the LGS Q-switched laser regime with a pulsed laser wavelength ranging from 1.0 μm–3.0 μm. The performance of the pulse laser with 200 kHz repetition rate is comparable to the BBO Q-switched laser with 200 kHz repetition rate^27^ and better than the KD^*^P (10 kHz)^18^, LN (7 kHz)^28^ and other LGS (30 kHz)^21^ Q-switched lasers. Additionally, the observed pulse width of 5.1 ns is also smaller than the narrowest pulse width obtained with KD^*^P (20 ns)^29^, LN (12 ns)^28^ and other LGS (7.8 ns)^21^ Q-switch. A RTP E-O Q-switched laser with the repetition rate of 280 kHz and pulse width 18.4 ns^7^ was reported, but a pair of RTP crystals should be used for compensating the natural birefringence due to the necessary of its orthogonal symmetry. Considering the achievable maximum repetition rate, the ease of crystal growth, the natural birefringence generated by the symmetry of the electro-optic crystal, the damage threshold (950 MW/cm^2 ^19), etc. As detailed shown in Table 2.

Compared with previous reported lasers with LGS as Q-switcher^21^, this present laser has some advantages in the repetition rates and pulse width which should be attributed to the shorter cavity length (185 mm) and lower applied electric voltage (3400 V). The shorter cavity determined the shorter roundtrip time and shorter pulse width and was constructed based on the “odd transit time” design which was proposed by the theoretical analysis on the optical active and electro-optic effects. The limitation of high repetition rates of an electro-optic Q-switched laser is the piezoelectric effect which would generate the “piezoelectric ring”. The LGS crystal has a small piezoelectric coefficient of 6 × 10^−12^ C/N, which indicates that the “piezoelectric ring” effect is negligible. Besides, the “piezoelectric ring” effect is also influenced by the applied electro-field. In the present experiments, we used a LGS Q-switcher with an large aspect ratio (5:1) which decreases the applied electro voltage of 3400 V lower than the previous best result in LGS Q-switched laser (3600 V)^21^. We believe that LGS crystal has great potential for application to the generation of high repetition rate laser radiation and could be used in higher power lasers. In the further, shortening the laser cavity and enlarging the aspect ratio should be helpful for the lasers with higher repetition rates and shorter pulse width which are favorable in many applications such as materials processing.

In conclusion, the interaction between optical activity and electro-optic property was theoretically analyzed and an “odd transit time” design was developed, which indicates that by rotating the quarter wave plate, the optical activity can be eliminated and the Q-switching performances can be realized when the light transits the Q-switch with odd times. Compared with the previous “even transit time” method related to the reversibility of the optical activity^30^, the present “odd transit time” design can simplify the configuration of the Q-switched laser cavity when using an optically active electro-optic crystal as the Q-switch. Using the “odd transit time” method and a Nd:LuVO_4_ crystal possessing a large emission cross-section and a short fluorescence lifetime as the gain medium, a LGS electro-optic Q-switched laser was constructed with a repetition rate of 200 kHz, average output power of 4.39 W and pulse width of 5.1 ns. These results indicate that LGS can be used as a high repetition rate Q-switch and free of piezoelectric ringing effects at least at a repetition rate of 200 kHz, and that it can provide a practical Q-switched laser with a tunable high repetition rate for many applications, such as materials processing, laser ranging, remote sensing, etc.

## Methods

### Theoretical analysis

Optical activity can be considered to be the birefringence of right-handed and left-handed circularly polarized light with refractive indices of n_R_ and n_L_^31^, respectively, where n_R_ and n_L_ can be expressed as:


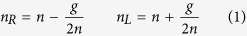


where n includes n_x_ and n_y_, the refractive indices along the X- and Y- directions as light propagates in an optically active crystal along the Z- direction.

The polarization state of right-handed and left-handed circularly polarized light can be expressed as^32^:


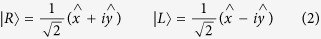


where 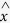 and 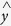 represent unit vectors along the X- and Y- axes, respectively. Therefore, for light with a polarization direction at an angle of 45° with the X-axis, the polarization state of the light is given as:


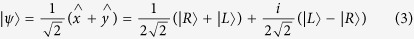


when the above light propagates in an optically active crystal along the Z-direction with length L and refractive index n_x_ and n_y_ along the X- and Y- directions, respectively, the polarization state can be expressed as:


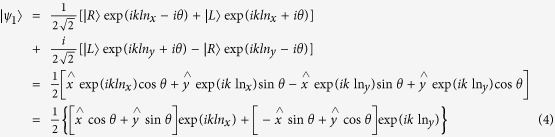


where: 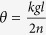, and g is the gyration tensor component of the optically active crystal.

To highlight the influence of optical activity on the birefringence, the polarization state can be simplified to:


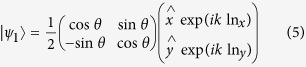


From eq. (5), we see that optical activity for propagation along the Z-axis involves only the polarization rotation direction with angle θ between left and right handed direction, based on Euler’s rotation theorem^33^. This result corresponds to a polarization rotation defined in an optically active process^34^ or the rotation of frames along the Z-axis with a rotation of θ from the right to the left-handed direction. This rotation would be eliminated when the light propagates along the -Z direction by taking advantage of the reversibility of optical activity^35^.

For a uniaxial optically active crystal, such as LGS, n_x_ = n_y_ = n_o_, where n_o_ is the refractive index of O-light. However, for LGS which has optically activity and electro-optic properties, when we apply an electric field E along the X-direction, the refractive indices along the X- and Y- axes are given by:


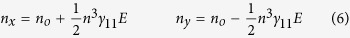


where: the birefringence is 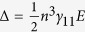
and the electro-optic coefficient is *γ*_11_. If E [used same symbol for electric field earlier] is the quarter-wave voltage, the rotation also indicates that a quarter wave plate should be rotated by an angle of θ in coincidence with the frame rotation. When the light is back-reflected, the frame rotation is eliminated and the polarization direction becomes parallel to that of the incident light, which means that the other components in the laser cavity are not influenced by the optical activity. Therefore, we conclude that the optical activity only induces frame rotation in the electro-optic crystal and the quarter wave plate rather than generating additional birefringence. By rotating the quarter wave plate, the electro-optic crystal with optical activity can generate electro-optic modulation similar to those crystals with no optical activity, such as KD^*^P^29^ and BBO^36^. Compared with the “even transit time”^30^ design, which is described as follows: the light propagates in the LGS crystal along the Z direction and then along the -Z direction in order to eliminate the effect of optical activity in the laser cavity^30^, the present analyzed “odd transit time” design requires only the rotation of a quarter wave plate thus simplifying the laser cavity configuration.

### Experiments of Q-switched laser

**Figure 3:**
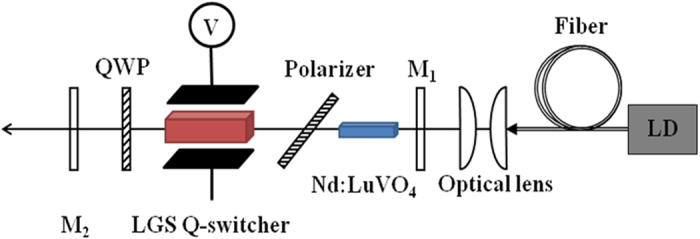
Experimental setup of the LGS electro-optic Q-switched Nd:LuVO_4_ laser.

Based on the above theoretical analysis, a straight cavity of “odd transit time” design as the following experiments were performed. A two-mirror straight cavity with a length of about 185 mm is employed and the configuration is shown in Fig. 3. The LGS electro-optic crystal was cut along the Z-axis with dimensions of 5 mm × 5 mm × 25 mm (X × Y × Z). The transmission surfaces were polished and AR coated at 1066 nm and the YZ surfaces were unpolished and coated with Au. The quarter wave voltage was calculated to be 3400 V based on a 5:1 aspect ratio. The crystal was rotated around the Z-axis, with the light polarization direction making an angle of 45° with the X-axis. The polarization direction is rotated to 27.5° after propagation through the LGS crystal and determined the rotation of the quarter wave plate (QWP) at 1066 nm at an angle of 27.5°. In other words, the optical activity has no influence on the polarization direction of the light with the rotated quarter wave plate as the light propagated through the Q-switch. The voltage was supplied with a homemade electro-driver with a maximum repetition rate of 200 kHz and a rise time of 8 ns. The pump source was a fiber-coupled laser diode with a central wavelength of 808 nm. The fiber diameter is 200 μm with a numerical aperture of 0.22. The pump light is focused onto the laser crystal by an imaging unit with a beam compression ratio of 1:1. The input mirror M_1_ is plane, antireflection (AR) coated for the pump wavelength and high-reflective (HR) coated for the laser wavelength at 1066 nm. The output coupler M_2_ is a plano-concave mirror with a radius of curvature of 200 mm and an output coupler (OC) transmission at 1066 nm of 15% or 10%. A Nd:LuVO_4_ crystal cut along the a-axis with a doping concentration of 0.4 at% was chosen as the laser crystal, since it has a large emission cross-section (14.6 × 10^−19^ cm^2^)^25^, short fluorescence lifetime (82 μs)^25^ and high conductivity (9.94 W/mK)^26^, all of which are beneficial for obtaining high repetition rate laser output with short pulse width and high peak power. The dimensions of the Nd:LuVO_4_ sample were 3 mm × 3 mm × 8 mm and the two 3 mm × 3 mm surfaces were polished and AR coated at 808 nm and 1066 nm. The polarizer was a quartz plate oriented along the Brewster angle. The average output power was measured by a power meter (1916-R, Newport, Inc.) and the temporal pulse behavior of the LGS Q-switched laser was recorded by a MSO3054 digital oscilloscope (500 MHz bandwidth and 2.5 GS/s sample rate, Tektronix, Inc.).

## Additional Information

**How to cite this article**: Ma, S. *et al*. Efficient high repetition rate electro-optic Q-switched laser with an optically active langasite crystal. *Sci. Rep.* **6**, 30517; doi: 10.1038/srep30517 (2016).

## Figures and Tables

**Table 1:** Summary of LGS electro-optic Q-switched Nd:LuVO_4_ crystal lasers with different repetition rate and OC = 15%.

Repetition rate(kHz)	Maximum average output power(W)	Minimum pulse width(ns)	Maximum single pulse energy(μJ)	Maximum peak power(kW)
200	4.39	5.1	21.95	1.7
150	4.46	9	29.75	2.0
100	4.11	8.6	41.12	4.48
50	4.07	9.7	81.56	6.5
20	3.88	9.3	194.35	16.3

**Table 2:** The performances of difference electro-optic Q-switched lasers.

Electro-optic Q-switch	LGS^1^	LN	KD^*^P	BBO	RTP	LGS^2^
Maximum high repetition rate (kHz)	30^21^	7^28^	10^18^	200^27^	280^7^	200
Minimum pulse width (ns)	7.8^21^	12^28^	20^29^	5^27^	18.4^7^	5.1
Piezoelectric coefficient (C/N)	—	23 × 10^−12 ^37	58 × 10^−12 ^38	26 × 10^−12 ^39	—	6 × 10^−12 ^24

LGS^1^ is the previous and LGS^2^ is the result of this work.

## References

[b1] Sun Y. L. . Protein-based three-dimensional whispering-gallery-mode micro-lasers with stimulus-responsiveness. Sci. Rep. 5, 12852 (2015). 26238255 10.1038/srep12852 PMC4523880

[b2] Tang Y. & Xu J. A random Q-switched fiber laser. Sci. Rep. 5, 9338 (2015). 25797520 10.1038/srep09338 PMC4369747

[b3] Chernysheva M. . High power Q-switched thulium doped fibre laser using carbon nanotube polymer composite saturable absorber. Sci. Rep. 6, 24220 (2016). 27063511 10.1038/srep24220 PMC4827123

[b4] Bernard J. E. & Alcock A. J. High-repetition-rate diode-pumped Nd:YVO_4_ slab laser. Opt. Lett. 19, 1861–1863 (1994). 19855678 10.1364/ol.19.001861

[b5] Liu Z. . Pulse-off electro-optic Q-switch made of La_3_Ga_5_SiO_14_. Opt. Express. 13, 7086–7090 (2005). 19498731 10.1364/opex.13.007086

[b6] Furuta K., Kojima T., Fujikawa S. & Nishimae J. Diode-pumped 1 kW Q-switched Nd: YAG rod laser with high peak power and high beam quality. Appl. Opt. 44, 4119–4122 (2005). 16004059 10.1364/ao.44.004119

[b7] Yu Y. J., Chen X. Y., Wang C., Wu C. T. & Jin G. Y. High repetition rate 880nm diode-directly-pumped electro-optic Q-switched Nd:GdVO_4_ laser with a double-crystal RTP electro-optic modulator. Opt. Commun. 304, 39–42 (2013).

[b8] Omatsu T., Isogami T., Minassian A. & Damzen M. J. >100kHz Q-switched operation in transversely diode-pumped ceramic Nd3 ^+^ :YAG laser in bounce geometry. Opt. Commun. 249, 531–537 (2005).

[b9] Hong H., Huang L., Liu Q., Yan P. & Gong M. Compact high-power, TEM_00_ acousto-optics Q-switched Nd:YVO_4_ oscillator pumped at 888 nm. Appl. Opt. 51, 323–327 (2012). 22270659 10.1364/AO.51.000323

[b10] Ji J., Zhu X., Dai S. & Wang C. Depolarization loss compensated resonator for electro-optic Q-switched solid-state laser. Opt. Commun. 270, 301–304 (2007).

[b11] Wang Z. . High-performance langasite (La_3_Ga_5_SiO_14_) electro-optic Q-switch. Opt. Laser. Technol. 39, 72–77 (2007).

[b12] Yu X. . Comparison of electro-optical and acousto-optical Q-switched, high repetition rate Nd:GdVO_4_ laser. Laser. Phys. 21, 442–445 (2011).

[b13] Volk T. R., Pryalkin V. I. & Rubinina N. M. Optical-damage-resistant LiNbO_3_:Zn crystal. Opt. Lett. 15, 996–998 (1990). 19770977 10.1364/ol.15.000996

[b14] Wang C., Zang H., Li X., Lu Y. & Zhu X. LD-pumped high repetition rate Q-switched Nd:YVO_4_ laser by using La_3_Ga_5_SiO_14_ single crystal electro-optic modulator. Chin. Opt. Lett. 4, 329–331 (2006).

[b15] Tsvetkov E. G., Khranenko G. G. & Solntsev V. P. General approaches to design of a reproducible technique for the growth of large crystals of barium metaborate (BBO) for industrial application. J. Cryst. Growth. 275, 2123–2128 (2005).

[b16] Dawes J. M. & Sceats M. G. A high repetition rate pico-synchronous Nd:YAG laser. Opt. Commun. 65, 275–278 (1988).

[b17] Hilbery R. P. & R. Hook W. Transient elastooptic effects and Q-switching performance in lithium niobate and KD^*^ P pockels cells. Appl. Opt. 9, 1939–1940 (1971). 10.1364/AO.9.001939 20094173

[b18] Gloster L. A. W., Cormont P., Cox A. M. & Chai B. H. H. Diode-pumped Q-switched Yb: S-FAP laser. Opt. Commun. 146, 177–180 (1998).

[b19] Li Y. . A novel La_3_Ga_5_SiO_14_ electro-optic Q-switched Nd:LiYF (Nd:YLF) laser with a cassegrain unstable cavity. Opt. Commun. 244, 333–338 (2005).

[b20] Kong H. K. . Growth, properties and application as an electro-optic Q-switch of langasite crystal. J. Cryst. Growth. 254, 360–367 (2003).

[b21] Tang H., Zhu X. & Feng Y. Comparison of 30 kHz Q-switched Nd:YVO_4_ lasers with LGS and RTP electro-optic modulator. Pacific Rim. 1, 1–4 (2009).

[b22] Wang L. . 520 mJ langasite electro-optically Q-switched Cr, Tm, Ho: YAG laser. Opt. Lett. 37, 1986–1988 (2012). 22660096 10.1364/OL.37.001986

[b23] Wang L. . 2.79 μm high peak power LGS electro-optically Q-switched Cr, Er: YSGG laser. Opt. Lett. 38, 2150–2152 (2013). 23939006 10.1364/OL.38.002150

[b24] Bohm J. . Czochralski growth and characterization of piezoelectric single crystals with langasite structure: La_3_Ga_5_SiO_14_ (LGS), La_3_Ga_5.5_Nb_0.5_O_14_ (LGN) and La_3_Ga_5.5_Ta_0.5_O_14_ (LGT) II. piezoelectric and elastic properties. J. Cryst. Growth. 216, 293–298 (2000).

[b25] Yu H. . Advances in vanadate laser crystals at a lasing wavelength of 1 micrometer. Laser. Photon. Rev. 8, 847–864 (2014).

[b26] Cheng Y. . Thermal properties and continuous-wave laser performance of Yb:LuVO_4_ crystal. Appl. Phys. B. 86, 681–685 (2007).

[b27] Nickel D. . 200 kHz electro-optic switch for ultrafast laser systems. Rev. Sci. Instrum. 76, 033111 (2005).

[b28] Chen Y. H. & Huang Y. C. Actively Q-switched Nd:YVO_4_ laser using an electro-optic periodically poled lithium niobate crystal as a laser Q-switch. Opt. Lett. 28, 1460–1462 (2003). 12943091 10.1364/ol.28.001460

[b29] Liu Q., Gong M., Wu H., Lu F. & Li C. Electro-optic Q-switched Yb:YAG slab laser. Laser. Phys. Lett. 3, 249 (2006).

[b30] Yin X., Wang J. & Zhang S. La_3_Ga_5_SiQ_14_ single-crystal Q-switch used as an electro-optic device. Appl. Opt. 42, 7188–7190 (2003). 14717297 10.1364/ao.42.007188

[b31] Wang Y., Zhang H., Yu H., Wang Z. & Wang J. Light propagation in an optically active plate with topological charge. Appl. Phys. Lett. 101, 171114 (2012).

[b32] Milione G., Sztul H. I., Nolan D. A. & Alfano H. R. R. igher-order poincaré sphere, stokes parameters, and the angular momentum of light. Phys. Rev. Lett. 107, 053601 (2011). 21867067 10.1103/PhysRevLett.107.053601

[b33] Weisstein E. W. Euler angles. Mathworld A Wolfram Web Resource. (2009).

[b34] Xi X. M. . Optical activity in twisted solid-core photonic crystal fibers. Phys. Rev. Lett. 110, 143903 (2013). 25166991 10.1103/PhysRevLett.110.143903

[b35] Vansteenkiste N., Vignolo P. & Aspect A. Optical reversibility theorems for polarization: application to remote control of polarization. J. Opt. Soc. Am. A. 10, 2240–2245 (1993).

[b36] Goodno G. D. . Investigation of β-BaB_2_O_4_ as a Q switch for high power applications. Appl. Phys. Lett. 66, 1575–1577 (1995).

[b37] Bhagavannarayana G., Budakoti G. C., Maurya K. K. & Kumar B. Enhancement of crystalline, piezoelectric and optical quality of LiNbO_3_ single crystals by post-growth annealing and poling. J. Cryst. Growth. 282, 394–401 (2005).

[b38] Sliker T. R. & Burlage S. R. Some dielectric and optical properties of KD_2_PO_4_. J. Appl. Phys. 34, 1837 (1963).

[b39] Andrushchak A. S. . Anisotropy of piezo- and elastooptical effect in β-BaB_2_O_4_ crystals. Ferroelectr. 238, 299–305 (2000).

